# Physical health trajectories of young people with neurodevelopmental conditions: a protocol for a systematic review of longitudinal studies

**DOI:** 10.1136/bmjopen-2024-090823

**Published:** 2025-04-27

**Authors:** Naomi Wilson, Ruchika Gajwani, Michael Fleming, Mia Findlay, Helen Stocks, Graham Walker, Neave Corcoran, Helen Minnis

**Affiliations:** 1 University of Glasgow, Glasgow, UK; 2 Institute of Health and Wellbeing, University of Glasgow, Glasgow, UK; 3 Public Health, University of Glasgow, Glasgow, UK; 4 General Practice and Primary Care, Institute of Health and Wellbeing, University of Glasgow, Glasgow, UK; 5 Mental Health and Wellbeing, University of Glasgow, Glasgow, UK

**Keywords:** PSYCHIATRY, Chronic Disease, PUBLIC HEALTH, Child & adolescent psychiatry

## Abstract

**Abstract:**

**Introduction:**

There is now emerging evidence to suggest a longitudinal association between specific neurodevelopmental conditions (NDCs) in childhood or adolescence (ie, autism, attention deficit hyperactivity disorder (ADHD) and tic disorders) and certain physical long-term conditions (LTCs) in adulthood. However, to date, this literature has never been comprehensively collated and appraised. As a result, our understanding of all the future health risks that young people with NDCs may collectively be at risk of is limited, and the factors which drive these adult health outcomes also remain obscure.

**Methods and analysis:**

A search strategy has been developed in collaboration with two medical librarians and will be used to conduct systematic searches of MEDLINE, EMBASE, APA PsycINFO, CINAHL and Web of Science. Prospective longitudinal studies exploring the association between three common NDCs in childhood or adolescence (ie, ADHD, autism and tic disorders <18 years of age) and any physical LTC in adulthood (ie, >18 years of age) will be selected through title and abstract review, followed by a full-text review. Data extracted will include the definition of exposure and outcome, mediators or moderators investigated, confounders adjusted for, and crude and adjusted effect estimates. Risk of bias assessment will be conducted. Results will be synthesised narratively and, if the data allow, a meta-analysis will also be conducted.

**Ethics and dissemination:**

Ethics approval is not applicable for this study since no original data will be collected. The results of the review will be widely disseminated locally, nationally and internationally through peer-reviewed publications, adhering to the Preferred Reporting Items for Systematic Reviews and Meta-Analyses statement, and conference presentations.

**PROSPERO registration number:**

CRD42024516684.

**STRENGTHS AND LIMITATIONS OF THIS STUDY**

- To our knowledge, this is the first systematic review synthesising and critically assessing evidence from longitudinal, observational studies on the prospective association between neurodevelopmental conditions (NDCs) in childhood or adolescence collectively and physical long-term conditions in adulthood.
- We will conduct a comprehensive search across multiple databases, without publication restrictions and will adhere to the Cochrane Handbook for Systematic Reviews of Interventions and the Preferred Reporting Items for Systematic Review and Meta-Analysis Protocols recommendations to ensure methodological rigour.
- This study’s focus on longitudinal evidence from observational studies will strengthen the conclusions drawn from results and may facilitate causal inference across studies.
- Depending on its findings, this study may represent a healthier sample of people with NDCs due to studies with significant loss to follow-up.
- We plan to meta-analyse outcome data; however, due to possible heterogeneity between studies, this may not be appropriate.

## Introduction

 The number of young people being diagnosed with a neurodevelopmental condition (NDC) has dramatically increased in recent years.^1^ Specifically, there has been a substantial upsurge in the number of children and adolescents being referred to mental health services for suspected attention deficit hyperactivity disorder (ADHD), autism and tic disorders (ie, Transient or Chronic Tic Disorders and Tourette’s Syndrome), without an associated intellectual disability.^2^,^6^ Although neurodevelopmental profiles change across the life course, it is widely recognised that these are lifelong conditions with implications extending into adulthood.^7^ As such, an accurate understanding of the future health needs of these young people is essential to informing the development and implementation of services and policies that address these.

It is now widely accepted that when left without appropriate support, young people with NDCs are at an increased risk of many of the social and psychiatric outcomes, which are known to be key drivers of physical health inequalities globally.^8 9^ Specifically, recent systematic reviews of longitudinal studies have shown that compared with the general population, young people with NDCs are more likely to experience a range of mental health difficulties in adulthood, including substance use, anxiety, and depressive disorders.^10 11^ Potentially partially due to these comorbidities, alongside a lack of educational support,^12^ having an NDC in childhood or adolescence can also increase the risk of future unemployment,^13^ socioeconomic disadvantage^14^ and social exclusion.^15^ However, despite knowledge of these heightened risks, until recently, relatively little research or clinical attention has been paid to the physical health trajectories that young people with NDCs may experience.

### Evidence before this study

It is well recognised that in childhood, NDCs are associated with a range of physical health conditions and complaints.^16^ Specifically, a systematic review of cross-sectional studies identified that children with NDCs are at an increased risk of autoimmune conditions, epilepsy and gastrointestinal complaints.^17^ A growing body of evidence is also now emerging to demonstrate a longitudinal association between specific NDCs in childhood and adolescence and certain physical long-term conditions (LTCs) in adulthood. For example, an increased risk of type II diabetes mellitus has recently been shown among adults diagnosed with Tourette’s Syndrome as children,^18^ while an increased risk of coronary artery disease has been identified among those previously diagnosed with ADHD.^19^ Epidemiological studies suggest that most people with these common chronic morbidities have at least two other, and frequently more, LTCs.^20^ It is also now increasingly understood that NDCs rarely occur in isolation and frequently co-occur.^21^ For instance, young people with autism are known to have a mean of 3.2 comorbid NDCs,^22^ and there is significant overlap in the aetiology, symptoms and consequences associated with these conditions.^23 24^

### Added value of the study

The risk of experiencing multiple physical LTCs in adulthood associated with experiencing any NDC in childhood and adolescence may therefore be substantial. However, whether this is the case remains unclear, as to date this emerging research area has been hindered by a selective focus on specific childhood NDCs or on particular physical health outcomes. Moreover, the emerging body of longitudinal research which does exist has never been systematically collated or appraised. As recently highlighted by Larsson and colleagues,^25^ a crucial first step in advancing our understanding of all of the future health risks that young people with NDCs face is therefore integrating the results of the existing longitudinal studies in this area.

Second, while some of the factors which may account for this association have been hypothesised, the exact mechanisms which underpin it are unclear and potential targets for early intervention are therefore obscure.^26^ These underlying mechanisms are likely complex, but modifiable lifestyle and social factors most probably play a role.^25^ Genetic and biological factors (ie, neuromodulatory systems) have also previously been implicated,^27 28^ but their role has never been systematically appraised. Another important task in furthering this research area is therefore identifying any biological, behavioural, psychosocial, material, structural or genetic factors which influence the risk of physical LTCs in NDCs across the life course. Comprehensively appraising the longitudinal studies which do exist is necessary in order to determine which of these factors have so far been conclusively implicated and to identify any remaining research or knowledge gaps on underlying mechanisms.

### Review objectives

The purpose of the current review is therefore to systematically collate and appraise the research which has been conducted to date exploring the longitudinal association between three common NDCs (ADHD, autism and tic disorders) in childhood or adolescence (ie, <18 years of age) and isolated or multiple physical LTCs in adulthood (ie, >18 years of age). In doing so, we aim to answer the following research questions.

(RQ1) Is there existing evidence to suggest a longitudinal association between ADHD, autism or tic disorders in childhood or adolescence and the subsequent development of isolated or multiple physical LTCs in adulthood?

(RQ2) Are there any modifiable factors (ie, biological, behavioural, psychosocial, material, structural or genetic) which have been shown to mediate or moderate this association?

For conditions where an appropriate body of homogeneous evidence exists, we will also conduct a meta-analysis to determine what the pooled estimate is, from the existing literature, regarding the risk of each condition or condition category in adulthood among those diagnosed with an included NDC in childhood or adolescence.

## Methods and analysis

The Preferred Reporting Items for Systematic Review and Meta-Analysis Protocols (PRISMA-P) recommendations have been used to guide the reporting in this systematic review protocol. We will follow the guidance provided in the Cochrane Handbook for Systematic Reviews of Interventions^29^ for conducting the review itself and the PRISMA statement (2020)^30^ for the reporting of our results.^31^ This systematic review protocol has been registered in the International Prospective Register of Systematic Reviews.

### Patient and public involvement

Patients and the public were involved in the development and scope of this review and will be involved in the reporting of its findings. This has been undertaken with the support of a working patient and public involvement and engagement (PPIE) group. We sought PPIE group members’ thoughts on the reviews objectives and included conditions during an initial focus group discussion. Based on their feedback during further focus groups, we will ensure that this review reports outcomes of relevance and importance to stakeholders and that the implications of our findings to the neurodivergent community are considered. Finally, we will also seek the guidance of PPIE group members prior to disseminating our findings, via a lay summary, peer-reviewed publication and conference presentations, to ensure translatability of results.

### Eligibility criteria

Study selection and eligibility criteria will be based on the population, exposure, comparison, outcome and study design (table 1).

**Table 1:** PECOS criteria

Population	Children and adolescents (<18 years old) with an included neurodevelopmental condition
Exposure	Three common neurodevelopmental conditions (ADHD, autism and/or tic disorders)
Comparison	Children and adolescents (<18 years old) without an included neurodevelopmental condition
Outcome	Physical long-term conditions (LTCs) in adulthood (>18 years old)
Study design	Longitudinal observational studies (ie, case–control or cohort)

ADHD, attention deficit hyperactivity disorder; PECOS, population, exposure, comparison, outcome, study design.

We will search for observational, longitudinal studies, from any country, which have explored the prevalence of isolated physical LTCs and/or multimorbidity among adults (ie, those over the age of 18) diagnosed with an included NDC in childhood or adolescence (ie, before the age of 18). Studies will subsequently be deemed eligible for inclusion if they adhere to the inclusion and exclusion criteria outlined below.

#### Inclusion criteria

1. Longitudinal observational studies exploring the association between an *incident* included NDC identified in childhood or adolescence (ie, before the age of 18) and any *subsequent* physical LTCs present in adulthood (ie, over the age of 18).
2. To minimise heterogeneity within our sample population, studies will be required to have adopted a systematic approach to the identification of NDC symptoms in childhood as defined by the relevant Diagnostic and Statistical Manual of Mental Disorders (DSM) or International Classification of Disease (ICD) criteria, or through the use of a research diagnostic protocol that matched these standards by considering symptom range, threshold, presence of impairment, chronicity and pervasiveness.
3. To ensure studies can be considered as beginning in childhood or adolescence, only those where participants had a mean age of <18 years old at baseline will be included.
4. To ensure studies are longitudinal in nature and that participants could reasonably be considered adults at follow-up, only studies where a proportion of participants were ≥ 18 years old at the time of assessment of our outcome of interest will be included.
5. The outcome conditions of interest are any physical LTCs (eg, cardiovascular disease, stroke, diabetes, painful musculoskeletal conditions, chronic obstructive pulmonary disease etc) occurring in adulthood (i.e., at or over the age of 18). As per the National Institute of Health Research recommendations, this will be defined as any health problem that requires ongoing management over a period of years and which cannot currently be cured but can be controlled with the use of medication and/or other therapies.
6. Studies which include any cancer as an outcome will also be included, as this is now widely considered to be an LTC.
7. Only studies which include a comparison sample of individuals without an NDC identified in childhood or adolescence, either through the inclusion of a reference population or a control group, will be included.
8. Publication in English in a book or peer-reviewed journal.

#### Exclusion criteria

1. Cross-sectional studies.
2. Studies which exclusively include those diagnosed with an included NDC in adulthood, based on either symptoms currently experienced or the retrospective reporting of childhood symptoms, will be excluded.
3. Studies which do not include a comparison to a non-exposed control group (ie, case series) will be excluded.
4. Interventional studies, including drug trials which primarily focus on the outcomes associated with a single medication or intervention for an included NDC, will be excluded.
5. As the interest of the current review is on the development of LTCs across the life course, studies which focus on incident or comorbid congenital conditions (ie, congenital cardiac malformations, cerebral palsy, phenylketonuria etc) will also be excluded.
6. Any studies which only report on physical outcomes which are not considered to meet the above definition of an LTC (ie, acute infections, accidents or injuries) will be excluded.
7. As our focus is on the physical health trajectories associated with our exposure of interest, studies which only report on psychiatric or mental health outcomes will also be excluded.

### Information sources

Databases searched electronically will be MEDLINE, PsycINFO, EMBASE, CINAHL Plus and Web of Science (WoS). We have selected MEDLINE, EMBASE and WoS databases based on recommendations for the Cochrane Handbook covering major health sciences topics. We also selected specialist subject databases CINAHL Plus and PsycINFO based on their relevance to the study objectives. WHO’s trials portal and clinicaltrials.gov will be searched to identify unpublished studies or studies still ongoing. We shall conduct a manual search of the reference lists and forward and backward citation of all included studies using the WoS database for any additional papers. We shall exclude studies that are published in systematic or literature reviews but shall also examine the reference lists of relevant reviews for additional candidate studies.

### Search strategy

The final search strategy was developed in collaboration with two medical librarians and is included in online supplemental appendix 1.

Our exposure of interest was common NDCs, and we therefore chose ADHD, autism and/or tic disorders to be the focus of this review. Search terms for the NDCs of interest were drawn from recent Cochrane reviews for these conditions.^32^,^34^

Our outcome of interest is any physical LTCs occurring in adulthood. Therefore, we used broad search terms for physical LTCs generally. However, in order for our search strategy to be as comprehensive as possible, we also included search terms for the four conditions which account for the greatest number of disability adjusted life years (DALYs) due to physical LTCs in high-income countries where the majority of NDC studies have been conducted to date (i.e., cardiovascular disease, stroke, diabetes, painful musculoskeletal conditions and chronic obstructive pulmonary disease).^35^ In addition, we included search terms for any other conditions which have been repeatedly identified as the most prevalent co-occurring physical LTCs among adults with an incident common mental health condition generally (hypertension, hyperlipidaemia, obesity, peptic ulcer disease, irritable bowel syndrome and chronic pain)^36^,^40^ or among those with autism, ADHD or a tic disorder specifically (asthma, atopic dermatitis, allergic rhinitis, acne, enthesopathy, migraine and epilepsy).^39^

Broad search terms for physical LTCs generally and for the majority of included LTCs were drawn from the most recent Cochrane review of multimorbidity.^41^ Specific search terms for the remaining LTCs were drawn from previously published reviews on chronic pain,^42 43^ epilepsy,^44^ obesity,^45^ allergic rhinitis,^46^ peptic ulcer disease,^47^ irritable bowel syndrome,^48^ atopic dermatitis^49^ and acne.^50^ Search terms for longitudinal studies were drawn from the British Medical Journal Best Practice Guidelines.^51^ These key terms will be combined with Boolean logic to search for relevant studies, and medical subject headings will be used to capture concepts and maximise the number of studies retrieved.

### Study records

#### Data management

Titles and abstracts of studies retrieved from each database search will be exported to EndNote (V.X9) for deduplication, and then imported to Rayyan software for screening.^52^ All identified manuscripts will be screened manually by one of the included authors, and artificial intelligence software will not be employed.

#### Study selection process

Following de-duplication, the titles and abstracts of all identified studies will be independently screened by two reviewers against the predefined inclusion and exclusion criteria to identify potentially relevant studies. Articles deemed potentially eligible during this initial screening will then be retrieved in full text for further assessment. Full-text articles will then be independently reviewed by two authors to confirm eligibility based on the inclusion criteria. Reasons for exclusion at each stage will be documented in a PRISMA flow diagram. Any disagreements surrounding the eligibility at either screening stage will be resolved through discussion or consultation with a third reviewer. This two-stage screening process will ensure a thorough and systematic approach to study selection.

#### Data extraction

Using a standardised data collection form, two reviewers will independently extract data from the eligible studies. For included studies, this will include the author, year of publication, country of origin, study design, sample size, a breakdown of total sample ethnicity and sex, mean age of sample at study start and end, and/or mean period of follow-up, definition of exposure (ie, NDC assessment method) and outcome (ie, physical LTC assessment method), mediators or moderators investigated, confounders adjusted for (if any), crude and adjusted effect estimates (with 95% CI) and estimates of subgroup analysis (if any) (eg, by differing forms of NDC). For studies where the mean age of the exposure and control groups are different, where possible we will calculate a pooled mean age of the total sample at the study start and report this in our results. We anticipate that for the majority of studies, the mean age of the exposure group at study entry and the mean age at NDC diagnosis will be the same. However, where these differ, we will extract and report both mean ages where possible. Where this is not possible, we will indicate whether the mean age reported in our results refers to the mean age at study entry or the mean age at NDC diagnosis. Discrepancies will be resolved by a third reviewer if necessary.

### Outcomes and prioritisation

Our primary outcome of interest will be the risk of physical LTCs in adulthood among those diagnosed with an NDC in childhood, and this will be prioritised. Our secondary outcome of interest is any factors which have been identified to mediate or moderate this association. Data on any mediators or moderators involved in this association will subsequently be extracted from studies which also include this and will be analysed separately.

### Risk of bias assessment

Quality assessment of the included studies will be conducted by two reviewers independently using the Newcastle-Ottawa Scale (NOS).^53^ The NOS is a widely used measure designed to assess the quality of observational studies, with versions available for both case–control and cohort studies. It is currently a tool recommended by the Cochrane Collaboration and assesses studies according to eight items, categorised into three dimensions: selection, comparability and outcome or exposure for cohort studies or case–control studies respectively. Studies can be graded up to 1 point for all items, except comparability which has the potential to score up to 2 points, giving a maximum possible score of 9. Studies will be rated from 0 to 9 according to this scale by both reviewers, and scores will subsequently be compared and discussed. Any discrepancies will be resolved by a third reviewer if necessary. The agreed upon total scores will then be used to indicate the overall quality of each included study, with those studies rating 0–2 being identified as of poor quality, 3–5 as of medium quality and 6–9 as of high quality.

### Data synthesis

We will tabulate key information (eg, sample characteristics, exposure and outcome characteristics) regarding the association between included NDCs and physical LTCs grouped by body systems, such as those outlined in table 2 (ie, cardiovascular, metabolic and endocrine, respiratory, gastrointestinal, musculoskeletal and dermatological etc) and perform a narrative synthesis regarding these associations. In this narrative synthesis, we will attempt to explore differences and similarities in outcomes between the included NDCs and will also explore the possibility of reporting results for different sociodemographic (eg, by ethnicity or sex) or other potentially high-risk subgroups (ie, looked after or accommodated children).

**Table 2:** Adult LTCs included in search terms

Body system (based on ICD-10)	Conditions included
Neurological	Chronic pain, migraine, epilepsy
Cardiovascular	Cardiovascular disease, cardiac arrhythmias, hypertension, stroke
Metabolic and endocrine	Hyperlipidaemia, obesity, diabetes mellitus
Respiratory	Asthma, allergic rhinitis, chronic obstructive pulmonary disease
Gastrointestinal	Peptic ulcer disease/dyspepsia, irritable bowel syndrome
Musculoskeletal	Enthesopathy
Dermatological	Atopic dermatitis, acne

ICD, International Classification of Disease; LTCs, long-term conditions.

We will perform a separate narrative synthesis of any identified mediating or moderating factors in this association and present these results separately. Specifically, we will categorise any factors which have been found to influence this association according to either one of the recognised pathways to inequalities in health^54^ or to any biological or genetic influences identified through our search (table 3).

**Table 3:** Potential causal mechanisms

Potential pathway	Potential mediating factors
Behavioural	High-risk health behaviours (including dietary habits, alcohol use, illicit substance use or tobacco smoking)
Psychosocial	Adverse childhood experiences or comorbid mental health difficulties
Material	Educational attainment or socioeconomic status
Structural	Access to health services and resources
Biological	Neural pathways, autonomic nervous system markers or inflammatory markers (C reactive protein, tumour necrosis factor alpha)
Genetic	Predisposing genetic factors (ie, SNPs) or epigenetic alterations

SNP, single nucleotide polymorphism.

Where possible and appropriate, meta-analyses will also be performed to calculate the overall pooled estimates of the relationship between the included NDCs combined and different physical LTCs in adulthood. Both crude and adjusted results will be displayed where possible using the generic inverse variance method. Adjustment will be based on the definition outlined in each identified study, and where possible, a hierarchy of adjustment will be created depending on the factors that are adjusted for. Studies that report similar adjustments will be analysed separately in crude and adjusted models to assess potential confounding among studies that reported adjusted estimates.

A fixed-effects model will be used where heterogeneity is low (I^2^ value of <50%), and a random-effects model where heterogeneity is high (I^2^ value of 50% or more) according to the Cochrane Handbook criteria.^29^ If appropriate, a funnel plot will be used to explore the existence of publication bias by visual inspection. We will also undertake an objective assessment of publication bias using Egger’s regression test. Asymmetry of the funnel plot and/or statistical significance of Egger’s regression test (p <0.05) will be indicative of publication bias.

### Confidence in cumulative evidence

At least two independent reviewers will assess the strength of the body of evidence (according to the following categories: high, moderate, low and very low) for each individual outcome using the five Grades of Recommendations, Assessment, Development and Evaluation (GRADE) considerations for downgrading the certainty of evidence (risk of bias, impression, inconsistency, indirectness and publication bias) and the three GRADE considerations for upgrading the certainty of evidence (large magnitude of effect, dose-response gradient and residual confounding decreasing the overall effect).^55^

## Supplementary material

10.1136/bmjopen-2024-090823 online supplemental file 1

## Data Availability

Data sharing not applicable as no datasets generated and/or analysed for this study.
