# Supplementary material for: Physical health trajectories of young people with neurodevelopmental conditions: a protocol for a systematic review of longitudinal studies
Source: BMJ Open. 2025 Apr 27;15(4):e090823. doi: 10.1136/bmjopen-2024-090823 (PMC12035448; doi:10.1136/bmjopen-2024-090823)
Supplement: online supplemental file 1 [file bmjopen-15-4-s001.pdf]

Appendix 1. Search Terms

	MEDLINE (Ovid)	EMBASE (Ovid)	PSYCINFO (EBSCO)	CINHAL (EBSCO)	Web Of Science (Core Collection)
**Search Category 1. Neurodevelopmental Disorder Search Terms**					
1	exp Attention Deficit Disorder with Hyperactivity/	exp attention deficit disorder/ or exp attention deficit hyperactivity disorder/	exp Attention Deficit Disorder with Hyperactivity/	MH "Attention Deficit Hyperactivity Disorder"	(TI=("Attention Deficit Hyperactivity Disorder")) OR AB=("Attention Deficit Hyperactivity Disorder")
2	(ADHD or ADDH or ADHS or "AD/HD" or HKD or TDAH).ti,ab.	(ADHD or ADDH or ADHS or "AD/HD" or HKD or TDAH).ti,ab.	(ADHD or ADDH or ADHS or "AD/HD" or HKD or TDAH).ti,ab.	TI ((ADHD or ADDH or ADHS or "AD/HD" or HKD or TDAH)) or AB ((ADHD or ADDH or ADHS or "AD/HD" or HKD or TDAH))	TI=((ADHD or ADDH or ADHS or "AD/HD" or HKD or TDAH))) OR AB=((ADHD or ADDH or ADHS or "AD/HD" or HKD or TDAH))
3	((attention* or behav*) adj3 (defic* or dysfunc* or disorder*)).ti,ab.	((attention* or behav*) adj3 (defic* or dysfunc* or disorder*)).ti,ab.	((attention* or behav*) adj3 (defic* or dysfunc* or disorder*)).ti,ab.	TI ((attention* or behav*) n2 (defic* or dysfunc* or disorder*)) or AB ((attention* or behav*) n2 (defic* or dysfunc* or disorder*))	TI=(((attention* or behav*) NEAR/2 (defic* or dysfunc* or disorder*)))) OR AB=(((attention* or behav*) NEAR/2 (defic* or dysfunc* or disorder*)))
4	((disrupt* adj3 disorder*) or (disrupt* adj3 behav*) or (defian* adj3 disorder*) or (defian* adj3 behav*)).ti,ab.	((disrupt* adj3 disorder*) or (disrupt* adj3 behav*) or (defian* adj3 disorder*) or (defian* adj3 behav*)).ti,ab.	((disrupt* adj3 disorder*) or (disrupt* adj3 behav*) or (defian* adj3 disorder*) or (defian* adj3 behav*)).ab,ti.	TI ((disrupt* n2 disorder*) or (disrupt* n2 behav*) or (defian* n2 disorder*) or (defian* n2 behav*)) or AB ((disrupt* n2 disorder*) or (disrupt* n2 behav*) or (defian* n2 disorder*) or (defian* n2 behav*))	AB ((disrupt* n2 disorder*) or (disrupt* n2 behav*) or (defian* n2 disorder*) or (defian* n2 behav*)) or TI ((disrupt* n2 disorder*) or (disrupt* n2 behav*) or (defian* n2 disorder*) or (defian* n2 behav*))
5	(impulsiv* or inattentiv* or inattention*).ti,ab.	(impulsiv* or inattentiv* or inattention*).ti,ab.	(impulsiv* or inattentiv* or inattention*).ab,ti.	TI (impulsiv* or inattentiv* or inattention*) or AB (impulsiv* or inattentiv* or inattention*)	TI=((impulsiv* or inattentiv* or inattention*))) or AB=((impulsiv* or inattentiv* or inattention*))
6	(hyperkin* or hyper adj kin*).ti,ab.	(hyperkin* or hyper adj kin*).ti,ab.	(hyperkin* or hyper adj kin*).ti,ab.	TI (hyperkin* or hyper n1 kin*) or AB (hyperkin* or hyper n1 kin*)	TI=((hyperkin* or hyper NEAR/1 kin*))) or AB=((hyperkin* or hyper NEAR/1 kin*))
7	(hyperactiv* or hyper adj activ*).ti,ab.	(hyperactiv* or hyper adj activ*).ti,ab.	(hyperactiv* or hyper adj activ*).ti,ab.	TI (hyperactiv* or hyper n1 activ*) or AB (hyperactiv* or hyper n1 activ*)	TI=((hyperactiv* or hyper NEAR/1 activ*))) or AB=((hyperactiv* or hyper NEAR/1 activ*))
8	exp Hyperkinesis/	exp hyperkinesis/	exp Hyperactivity/	(MH "Hyperkinesis")	(TI=Hyperkinesis or AB=Hyperkinesis)
9	(minimal adj brain adj3 disorder*).ti,ab.	(minimal adj brain adj3 disorder*).ti,ab.	(minimal adj brain adj3 disorder*).ab,ti.	TI (minimal n2 brain n2 (disorder*)) or AB (minimal n2 brain n2 (disorder*))	TI=(minimal NEAR/1 brain NEAR/1 (disorder*)) or AB=(minimal NEAR/1 brain NEAR/1 (disorder*))
10	(minimal adj brain adj3 dysfunction*).ti,ab.	(minimal adj brain adj3 dysfunction*).ti,ab.	(minimal adj brain adj3 dysfunction*).ab,ti.	TI (minimal n2 brain n2 (dysfunct*)) or AB (minimal n2 brain n2 (dysfunct*))	TI=(minimal NEAR/1 brain NEAR/1 (dysfunct*)) or AB=(minimal NEAR/1 brain NEAR/1 (dysfunct*))
11	(minimal adj brain adj3 damage*).ti,ab.	(minimal adj brain adj3 damage*).ti,ab.	(minimal adj brain adj3 damage*).ab,ti.	TI (minimal n2 brain n2 (damage*)) OR AB (minimal n2 brain n2 (damage*))	TI=(minimal NEAR/1 brain NEAR/1 (damage*)) or AB=(minimal NEAR/1 brain NEAR/1 (damage*))
12	exp Child Development Disorders, Pervasive/ or exp Autistic Disorder/	exp autism/	exp Autism Spectrum Disorders/	MH "Child Development Disorders, Pervasive+"	(TI=("Child Development Disorders")) OR AB=("Child Development Disorders")
13	pervasive development* disorder*.ti,ab.	pervasive development* disorder*.ti,ab.	pervasive development* disorder*.ti,ab.	AB (“pervasive development* disorder*”) or TI (“pervasive development* disorder*”)	(TI=((“pervasive development* disorder*”))) OR AB=((“pervasive development* disorder*”))
14	(PDD or PDDs or ASD or ASDs).ti,ab.	(PDD or PDDs or ASD or ASDs).ti,ab.	(PDD or PDDs or ASD or ASDs).ti,ab.	AB (PDD or PDDs or ASD or ASDs) or TI (PDD or PDDs or ASD or ASDs)	(TI=((PDD or PDDs or ASD or ASDs))) OR AB=((PDD or PDDs or ASD or ASDs))
15	autis*.ti,ab.	autis*.ti,ab.	autis*.ti,ab.	TI (autis*) or AB (autis*)	(TI=(autism)) OR AB=(autism)
16	asperger*.ti,ab.	asperger*.ti,ab.	asperger*.ti,ab.	TI (asperger*) or AB (asperger*)	(TI=((asperger*))) OR AB=((asperger*))
17	kanner*.ti,ab.	kanner*.ti,ab.	kanner*.ti,ab.	TI (kanner*) or AB (kanner*)	(TI=(kanner*)) OR AB=(kanner*)
18	childhood schizophreni*.ti,ab.	childhood schizophreni*.ti,ab.	childhood schizophreni*.ti,ab.	TI (“childhood schizophren*”) or AB (“childhood schizophren*”)	AB (“childhood schizophren*”) or TI (“childhood schizophren*”)
19					
20	exp Tourette Syndrome/ or Tourette*.ti,ab.	exp Gilles de la Tourette syndrome/ or Tourette*.ti,ab.	exp Tourette Syndrome/ or Tourette*.ti,ab.	MH "Tourette Syndrome" or TI (Tourette*) or AB (Tourette*)	TI=(Tourette*) or AB=(Tourette*)
21	exp Tics/ or exp Tic Disorders/ or (tic or tics).ti,ab.	exp tic/ or (tic or tics).ti,ab.	exp Tics/ or exp Tic Disorders/ or (tic or tics).ti,ab.	MH “Tic” or TI (tic or tics) or AB (tic or tics)	AB (tic*) or TI (tic*)
**Search Category 2. Physical LTC Search Terms**					
22	exp Chronic Disease/	exp chronic disease/	exp Chronic Illness/	(MH "Chronic Disease+")	(TI=(chronic* NEAR/3 disease*)) OR AB=(chronic* NEAR/3 disease*)
23	((chronic* or "long term") adj3 (illness or disease* or disorder* or condition*)).ti,ab.	((chronic* or "long term") adj3 (illness or disease* or disorder* or condition*)).ti,ab.	((chronic* or "long term") adj3 (illness or disease* or disorder* or condition*)).ti,ab.	TI ((chronic* or "long term" or "long-term") n3 (illness or disease* or disorder* or condition*)) or AB ((chronic* or "long term" or "long-term") n3 (illness or disease* or disorder* or condition*))	TI=(("long term" or "long-term") NEAR/3 (illness or disease* or disorder* or condition*)) or AB=(("long term" or "long-term") NEAR/3 (illness or disease* or disorder* or condition*))
24	exp Epilepsy/	exp Epilepsy/	exp Epilepsy/	MH “Epilepsy”	(TI=(epilep*)) OR AB=(epilep*)
25	exp Seizures/	exp Seizures/	exp Seizures/	MH “Seizures”	(TI=(seizure*)) OR AB=(seizure*)
26	(epilep* or seizure* or convuls*).ti,ab.	(epilep* or seizure* or convuls*).ti,ab.	(epilep* or seizure* or convuls*).ti,ab.	TI (epilep* or seizure* or convuls*) or AB (epilep* or seizure* or convuls*)	(TI=(convuls*)) OR AB=(convuls*)
27	exp pain/	exp pain/	exp pain/	MH “pain”	-

28	(chronic adj2 pain*).ti,ab.	(chronic adj2 pain*).ti,ab.	(chronic adj2 pain*).ti,ab.	TI (chronic n2 pain*) or AB (chronic n2 pain*)	AB (chronic NEAR/2 pain*) or TI (chronic NEAR/2 pain*)
29	(chronic adj2 (discomfort or ache* or neuralgi*)).ti,ab.	(chronic adj2 (discomfort or ache* or neuralgi*)).ti,ab.	(chronic adj2 (discomfort or ache* or neuralgi*)).ti,ab.	TI (chronic n2 (discomfort or ache* or neuralgi*)) or AB (chronic n2 (discomfort or ache* or neuralgi*))	AB (chronic NEAR/2 (discomfort or ache* or neuralgi*)) or TI (chronic NEAR/2 (discomfort or ache* or neuralgi*))
	exp headache/ or exp migraine/	exp headache/ or exp migraine/	exp headache/ or exp migraine/		-
30	(headache* or migraine* or neuralgia*).ti,ab.	(headache* or migraine* or neuralgia*).ti,ab.	(headache* or migraine* or neuralgia*).ti,ab.	TI (headache* or migraine* or neuralgia*) or AB (headache* or migraine* or neuralgia*)	(TI=((headache* or migraine* or neuralgia*))) OR AB=((headache* or migraine* or neuralgia*))
31	exp heart diseases/ or exp cardiovascular disease/	exp heart disease/ or exp cardiovascular disease/	exp Heart Disorders/	MH "Heart Diseases+" or MH “cardiovascular disease”	-
32	(((heart or cardiac or cardiovascular or coronary) adj (disease* or disorder* or failure)) or arrythmia*).ti,ab.	(((heart or cardiac or cardiovascular or coronary) adj (disease* or disorder* or failure)) or arrythmia*).ti,ab.	(((heart or cardiac or cardiovascular or coronary) adj (disease* or disorder* or failure)) or arrythmia*).ti,ab.	TI (((heart or cardiac or cardiovascular or coronary) n1 (disease* or disorder* or failure)) or arrythmia*) or AB (((heart or cardiac or cardiovascular or coronary) n1 (disease* or disorder* or failure)) or arrythmia*)	(TI=((((heart or cardiac or cardiovascular or coronary) NEAR/1 (disease* or disorder* or failure)) or arrythmia*))) OR AB=((((heart or cardiac or cardiovascular or coronary) NEAR/1 (disease* or disorder* or failure)) or arrythmia*))
33	exp hypertension/	exp hypertension/	exp hypertension/	MH Hypertension	-
34	(hypertens* or "high blood pressure*").ti,ab.	(hypertens* or "high blood pressure*").ti,ab.	(hypertens* or "high blood pressure*").ti,ab.	TI (hypertens* or "high blood pressure*") or AB (hypertens* or "high blood pressure*")	(TI=((hypertens* or "high blood pressure*"))) OR AB=((hypertens* or "high blood pressure*"))
35	exp stroke/	exp cerebrovascular accident/ or exp stroke/	exp Cerebrovascular Accidents/	MH stroke	-
36	(stroke or strokes or cva or "cerebrovascular accident").ti,ab.	(stroke or strokes or cva or "cerebrovascular accident").ti,ab.	(stroke or strokes or cva or "cerebrovascular accident").ti,ab.	TI ((stroke or strokes or cva or "cerebrovascular accident")) or AB ((stroke or strokes or cva or "cerebrovascular accident"))	(TI=(((stroke or strokes or cva or "cerebrovascular accident")))) OR AB=(((stroke or strokes or cva or "cerebrovascular accident")))
37	exp diabetes mellitus/	exp diabetes mellitus/	exp diabetes mellitus/	MH diabetes mellitus	-
38	diabet*.ti,ab.	diabet*.ti,ab.	diabet*.ti,ab.	TI (diabet*) or AB (diabet*)	(TI=(diabet*)) OR AB=(diabet*)
39	exp hyperlipidemia/	exp hyperlipidemia/	exp lipid metabolism disorders/	MH hyperlipidemia	-
40	(hyperlipidem* or hypercholesterolemia* or hypertriglyceridemia*).ti,ab.	(hyperlipidem* or hypercholesterolemia* or hypertriglyceridemia*).ti,ab.	(hyperlipidem* or hypercholesterolemia* or hypertriglyceridemia*).ti,ab.	TI (hyperlipidem* or hypercholesterolemia* or hypertriglyceridemia*) or AB (hyperlipidem* or hypercholesterolemia* or hypertriglyceridemia*)	(TI=((hyperlipidem* or hypercholesterolemia* or hypertriglyceridemia*))) OR AB=((hyperlipidem* or hypercholesterolemia* or hypertriglyceridemia*))
41	exp Obesity/ or exp Obesity, Morbid/	exp obesity/ or exp morbid obesity/	exp Obesity/	(MH "Obesity+") OR (MH "Obesity, Morbid")	-
42	(adipos* or obes*).ti,ab.	(adipos* or obes*).ti,ab.	(adipos* or obes*).ti,ab.	TI (adipos* or obes*) or AB (adipos* or obes*)	(TI=((adipos* or obes*))) OR AB=((adipos* or obes*))
43	exp asthma/	exp asthma/	exp Asthma/	(MH "Asthma+")	-
44	asthma*.ti,ab.	asthma*.ti,ab.	asthma*.ti,ab.	TI (asthma*) or AB (asthma*)	(TI=(asthma*)) OR AB=(asthma*))
45	exp pulmonary disease chronic obstructive/	exp chronic obstructive lung disease/	exp Chronic Obstructive Pulmonary Disease/	(MH "Pulmonary Disease, Chronic Obstructive")	(TI=("Chronic Obstructive Pulmonary Disease")) OR AB=("Chronic Obstructive Pulmonary Disease"))
46	(copd or (pulmonary adj2 (disease* or disorder*))).ti,ab.	(copd or (pulmonary adj2 (disease* or disorder*))).ti,ab.	(copd or (pulmonary adj2 (disease* or disorder*))).ti,ab.	TI (copd or (pulmonary n2 (disease* or disorder*))) or AB (copd or (pulmonary n2 (disease* or disorder*)))	(TI=((copd or (pulmonary NEAR/2 (disease* or disorder*))))) OR AB=((copd or (pulmonary NEAR/2 (disease* or disorder*))))
47	exp allergic rhinitis/ or exp rhinitis/	exp allergic rhinitis/ or exp rhinitis/	exp Allergic Disorders/	MH “Rhinitis”	-
48	rhinit*.ti,ab.	Rhinit*.ti,ab.	Rhinit*.ti,ab.	TI (Rhinit*) or AB (Rhinit*)	(TI=(Rhinit*)) OR AB=(Rhinit*)
49	exp dyspepsia/ or exp peptic ulcer/ or exp stomach ulcer/	exp dyspepsia/ or exp peptic ulcer/ or exp stomach ulcer/	exp Dyspepsia/ or exp Gastrointestinal Ulcers/	(MH "Peptic Ulcer+") or (MH "Dyspepsia")	-
50	(dyspep* or (acid adj5 reflux)).ti,ab.	(dyspep* or (acid adj5 reflux)).ti,ab.	(dyspep* or (acid adj5 reflux)).ti,ab.	TI (dyspep* or (acid n4 reflux)) or AB (dyspep* or (acid n4 reflux))	(TI=((dyspep* or (acid NEAR/4 reflux)))) OR AB=((dyspep* or (acid NEAR/4 reflux)))
51	((pep* adj5 ulcer*) or (stomach adj5 ulcer*) or (gastr* adj5 ulcer*)).ti,ab.	((pep* adj5 ulcer*) or (stomach adj5 ulcer*) or (gastr* adj5 ulcer*)).ti,ab.	((pep* adj5 ulcer*) or (stomach adj5 ulcer*) or (gastr* adj5 ulcer*)).ti,ab.	TI ((pep* n4 ulcer*) or (stomach n4 ulcer*) or (gastr* n4 ulcer*)) or AB ((pep* n4 ulcer*) or (stomach n4 ulcer*) or (gastr* n4 ulcer*))	(TI=(((pep* NEAR/4 ulcer*) or (stomach NEAR/4 ulcer*) or (gastr* NEAR/4 ulcer*)))) OR AB=(((pep* NEAR/4 ulcer*) or (stomach NEAR/4 ulcer*) or (gastr* NEAR/4 ulcer*)))
52	exp Irritable Bowel Syndrome/	exp Irritable Bowel Syndrome/	exp Irritable Bowel Syndrome/	MH “irritable bowel syndrome”	(TI=(“irritable bowel syndrome”)) OR AB=(“irritable bowel syndrome”)
53	((irritable or functional or spastic) adj1 (bowel or colon)).ti,ab.	((irritable or functional or spastic) adj1 (bowel or colon)).ti,ab.	((irritable or functional or spastic) adj1 (bowel or colon)).ti,ab.	TI ((irritable or functional or spastic) n1 (bowel or colon))) or AB ((irritable or functional or spastic) n1 (bowel or colon)))	(TI=(((irritable or functional or spastic) NEAR/1 (bowel or colon)))) OR AB=(((irritable or functional or spastic) NEAR/1 (bowel or colon)))
54	ibs.ti,ab.	ibs.ti,ab.	ibs.ti,ab.	TI ibs or AB ibs	(TI=(ibs)) OR AB=(ibs)
55	exp Fibromyalgia/	exp Fibromyalgia/	exp Fibromyalgia/	MH fibromyalgia	-
56	fibromyalgia*.ti,ab	fibromyalgia*.ti,ab	fibromyalgia*.ti,ab	TI fibromyalgia* or AB fibromyalgia*	(TI=(fibromyalgia*)) OR AB=(fibromyalgia*)
57	Enthesopathy/	Enthesopathy/	exp joint disorders/	MH enthesopathy	(TI=(enthesopathy)) OR AB=(enthesopathy)
58	((musculoskeletal or joint*) adj3 pain*).ti,ab.	((musculoskeletal or joint*) adj3 pain*).ti,ab.	((musculoskeletal or joint*) adj3 pain*).ti,ab.	TI ((musculoskeletal or joint*) n2 pain*) or AB ((musculoskeletal or joint*) n2 pain*)	TI=(((musculoskeletal or joint*) NEAR/2 pain*))) OR AB=(((musculoskeletal or joint*) NEAR/2 pain*))
59	exp musculoskeletal pain/	exp musculoskeletal pain/	exp musculoskeletal disorders/	(MH "Musculoskeletal Pain") or (MH "Back Pain")	-
60	exp Dermatitis, Atopic/	exp atopic dermatitis/	exp Dermatitis/	(MH "Dermatitis, Atopic")	-
61	exp eczema/	exp eczema/	exp Eczema/	(MH "Eczema")	-

62	(atopic adj3 dermatit*).ti,ab.	(atopic adj3 dermatit*).ti,ab.	(atopic adj3 dermatit*).ti,ab.	TI (atopic n2 dermatit*) or AB (atopic n2 dermatit*)	(TI=((atopic NEAR/2 dermatit*))) OR AB=((atopic NEAR/2 dermatit*))
63	eczema.ti,ab.	eczema.ti,ab.	eczema.ti,ab.	TI (eczema) or AB (eczema)	TI=(eczema)) OR AB=(eczema)
64	exp acne vulgaris/ or acne.ti,ab.	exp acne vulgaris/ or acne.ti,ab.	acne.ti,ab.	(MH "acne vulgaris") or TI (acne) or AB (acne)	TI=(acne)) OR AB=(acne)
**Search Category 3. Longitudinal Studies**					
65	exp cohort studies/ or cohort$.tw.	exp cohort analysis/ or cohort$.tw.	exp Cohort Analysis/ or cohort$.tw.	TI ((cohort n3 stud*) or (cohort n1 analysis)) or AB ((cohort n3 stud*) or (cohort n1 analysis))	(((TI=(((cohort NEAR/3 stud*) or (cohort NEAR/1 analysis)))) OR AB=(((cohort NEAR/3 stud*) or (cohort NEAR/1 analysis))))
	(longitudinal adj3 stud*).ti,ab.	exp longitudinal study/ or (longitudinal adj3 stud*).ti,ab.	exp Longitudinal Studies/ or (longitudinal adj3 stud*).ti,ab.	TI ((longitudinal n3 stud*) or (longitudinal n1 research)) or AB ((longitudinal n3 stud*) or (longitudinal n1 research))	TI=(((longitudinal NEAR/3 stud*) or (longitudinal n1 research)))) OR AB=(((longitudinal NEAR/3 stud*) or (longitudinal n1 research)))
66	(prospective adj3 stud*).ti,ab.	exp prospective study/ or (prospective adj3 stud*).ti,ab.	exp Prospective Studies/ or (prospective adj3 stud*).ti,ab.	(MH "Prospective Studies+")	TI=((prospective NEAR/3 stud*))) OR AB=((prospective NEAR/3 stud*))
68	(follow-up adj3 stud*).ti,ab.	exp follow up/ or (follow-up adj3 stud*).ti,ab	(follow-up adj3 stud*).ti,ab.	TI (("follow up" n2 stud*) or (follow-up n2 stud*)) or AB (("follow up" n2 stud*) or (follow-up n2 stud*))	TI=((("follow up" NEAR/2 stud*) or (follow-up NEAR/2 stud*)))) OR AB=((("follow up" NEAR/2 stud*) or (follow-up NEAR/2 stud*)))
69	epidemiologic methods/ or controlled clinical trial.pt.	-	-	(MH "Nonexperimental Studies+") OR (MH "Quasi-Experimental Studies+")	

**Within category search terms combined with 'OR' and between category search terms combined with 'AND'.
