# Supplementary material for: Physical health trajectories of young people with neurodevelopmental conditions: a protocol for a systematic review of longitudinal studies
Source: BMJ Open. 2025 Apr 27;15(4):e090823. doi: 10.1136/bmjopen-2024-090823 (PMC12035448; doi:10.1136/bmjopen-2024-090823)
Supplement: Supplementary file 2 [file bmjopen-2024-090823.reviewer_comments.pdf]

PEER REVIEW HISTORY

BMJ Open publishes all reviews undertaken for accepted manuscripts. Reviewers are asked to complete a checklist review form and are provided with free text boxes to elaborate on their assessment. These free text comments are reproduced below.

ARTICLE DETAILS

Title (Provisional)

The Physical Health Trajectories of Young People with Neurodevelopmental Conditions: A Protocol for a Systematic Review of Longitudinal Studies.

Authors

Wilson, Naomi; Gajwani, Ruchika; Fleming, Michael; Findlay, Mia; Stocks, Helen; Walker, Graham; Corcoran, Neave; Minnis, Helen

---

VERSION 1 - REVIEW

**Reviewer**	**1**
**Name**	**Eccles, Jessica**
**Affiliation**	**Brighton and Sussex Medical School, Neuroscience**
**Date**	**24-Sep-2024**
**COI**	**I am writing an editorial with two of the authors and have previously submitted a grant application with one of them**

A timely review on an important topic. The strategy is sound

**Reviewer**	**2**
**Name**	**Lee, Alice**
**Affiliation**	**University College Cork, Department of Speech and Hearing Sciences**
**Date**	**15-Nov-2024**
**COI**	**None**

Introduction:

• Page 6 of 23, Line 13-15: suggest rephrasing the part, “many of these conditions were previously conceptualized as being limited to childhood”. Any health care professions and teachers who have worked with these individuals as well as their parents would know it is no limited to childhood (although the issues / concerns might change / shift over time). It is

probably better to say that relatively more is known about the conditions and associated issues for individuals in their childhood than in the adulthood.

Methods and analysis:

• P8, L18-23: suggest referencing to a systematic review manual for the methodology and refer to those two papers about following the guidelines about what to report in a systematic review.

• P8, L43: how will the PPIE group members be involved in the evidence synthesis stage? What will they be asked to do?

• P9, L10: does that mean the individuals who got the diagnosis before 18 years of age (as stated in the first inclusion criterion)?

• P9, L38-41: just want to get a clarification that does criterion #4 mean that studies that did not include longitudinal observations that go beyond age 18 will not be included?

• P9, L43: suggest giving examples of "physical LTCs" here; that is, moving the examples in L25 on P10 to here.

• P10, L34-36: the names (or the format) of some of the databases are slightly different in the Abstract e.g. "Medline" in the Abstract and "MEDLINE" in the Method section. Please revised.

• P10, L44: use "WoS" in this line as it is being used already in L36.

• P11, L59: Rayyan software seems to involve the use of AI for systematic review, and I am not sure whether the Journal has any policy or requirement of declaration around the use of AI in research. Would appreciate advice from the Editor.

• P12, L8-10: the step "full text of all remaining potentially eligible studies will be independently screened by two authors thereafter" is a little unclear. Is it only for those studies of which the eligibility cannot be decided based on "titles and abstracts" (in L7), or is it repeating the step stated in L7-9 i.e. screen all studies based on "titles and abstracts" first and then screen all studies again based on the full text?

• P12, L23: how about the age of NDC diagnosis if it was different from the "mean age of sample at study start".

• P12, L28: what kind of subgroups do you anticipate?

• P13, L21: will the "socio-demographic" info be extracted as well? Suggest stating that in the "Data extraction" section on P12.

• P13, L29: revise "to influences".

• P14, L8-11: suggest joining this very short paragraph with the previous one.

VERSION 1 - AUTHOR RESPONSE

Reviewer: 1
Dr. Jessica Eccles, Brighton and Sussex Medical School, Sussex Partnership NHS Foundation Trust

Comments to the Author:
A timely review on an important topic. The strategy is sound.

Response:
Many thanks for taking the time to review our manuscript and for providing your feedback, it is greatly appreciated.

Reviewer: 2: Dr. Alice Lee, University College Cork

Many thanks for taking the time to review our manuscript and for providing your helpful feedback on areas for adjustment and improvement. We have responded to each of your comments with details of the adaptions we have made to account for these.

Comments to the Author:
Introduction:

(1) Page 6 of 23, Line 13-15: suggest rephrasing the part, "many of these conditions were previously conceptualized as being limited to childhood". Any health care professions and teachers who have worked with these individuals as well as their parents would know it is no limited to childhood (although the issues / concerns might change / shift over time). It is probably better to say that relatively more is known about the conditions and associated issues for individuals in their childhood than in the adulthood.

- Thank you for highlighting this, we have now changed this sentence to: "Although neurodevelopmental profiles change across the life course, it is widely recognised that these are lifelong conditions with implications extending into adulthood"

Methods and Analysis:

• P8, L18-23: suggest referencing to a systematic review manual for the methodology and refer to those two papers about following the guidelines about what to report in a systematic review.

As there is currently no widely accepted manual for conducting systematic reviews of observational studies which we are aware of, we have now referenced the Cochrane Handbook of Systematic Reviews of Interventional Studies as our guideline for conducting the review itself and the PRISMA checklist for guiding the reporting of our results.

• P8, L43: how will the PPIE group members be involved in the evidence synthesis stage? What will they be asked to do?

We had envisioned meeting with our PPIE group to discuss the implications of our findings to the neurodivergent community and including this as part of our discussion (for example, if young people are at an increased risk of certain physical LTCs in adulthood, should this information be offered at the point of diagnosis? Are there any interventions they feel would be particularly helpful? etc.). However, on reflection this is not captured by the term 'evidence synthesis', and this sentence could therefore be unintentionally misleading. We have therefore altered our PPIE section so that it now reads:

"Patients and the public were involved in the development and scope of this review and will be involved in the reporting of its findings. This has been undertaken with the support of a working patient and public involvement and engagement (PPIE) group. We sought PPIE group members thoughts on the reviews objectives and included conditions during an initial focus group discussion. Based on their feedback during further focus groups, we will ensure that this review reports outcomes of relevance and importance to stakeholders and that the implications of our findings to the neurodivergent community are considered. Finally, we will also seek the guidance of PPIE group members prior to disseminating our findings, via a lay summary, peer reviewed publication and conference presentations, to ensure translatability of results."

• P9, L10: does that mean the individuals who got the diagnosis before 18 years of age (as stated in the first inclusion criterion)?

Yes. To clarify this, we have now rephrased this sentence to *"We will search for observational, longitudinal studies, from any country, which have explored the prevalence of isolated physical LTCs and/or multimorbidity among adults (i.e. those over the age of 18) diagnosed with an included NDC in childhood or adolescence (i.e., before the age of 18)."*

• P9, L38-41: just want to get a clarification that does criterion #4 mean that studies that did not include longitudinal observations that go beyond age 18 will not be included?

Yes, studies that do not include outcomes (physical LTCs) as assessed after the age of 18 will be excluded. To clarify this, we have now rephrased criterion #4 so that it reads *"To ensure studies are longitudinal in nature and that participants could reasonably be considered adults at follow-up, only studies where a proportion of participants were greater than 18 years old at the time of assessment of our outcome of interest will be included."*

• P9, L43: suggest giving examples of "physical LTCs" here; that is, moving the examples in L25 on P10 to here.

Several examples of physical LTCs have now been included here as suggested.

• P10, L34-36: the names (or the format) of some of the databases are slightly different

in the Abstract e.g. “Medline” in the Abstract and “MEDLINE” in the Method section. Please revised.

Thank you for highlighting this, all instances of this have now been corrected to MEDLINE.

• P10, L44: use “WoS” in this line as it is being used already in L36.

This change has now been made as suggested.

• P11, L59: Rayyan software seems to involve the use of AI for systematic review, and I am not sure whether the Journal has any policy or requirement of declaration around the use of AI in research. Would appreciate advice from the Editor.

While the Rayyan platform does have the potential to use AI software to assist with the screening process, this will not be employed by this systematic review. All identified articles will be screened manually by two of the included authors. We have now added a sentence to this paragraph to make this clear. It states: *“All identified manuscripts will be screened manually by two of the included authors, as outlined below, and Artificial Intelligence software will not be employed.”*

• P12, L8-10: the step “full text of all remaining potentially eligible studies will be independently screened by two authors thereafter” is a little unclear. Is it only for those studies of which the eligibility cannot be decided based on “titles and abstracts” (in L7), or is it repeating the step stated in L7-9 i.e. screen all studies based on “titles and abstracts” first and then screen all studies again based on the full text?

On reflection we agree that this paragraph was not as clear as it could have been regarding the screening process. It has therefore been reworded for clarity and now reads: *“Following de-duplication, the titles and abstracts of all identified studies will be independently screened by two reviewers against the predefined inclusion and exclusion criteria to identify potentially relevant studies. Articles deemed potentially eligible during this initial screening will then be retrieved in full text for further assessment. Full-text articles will then be independently reviewed by two authors to confirm eligibility based on the inclusion criteria. Reasons for exclusion at each stage will be documented in a PRISMA flow diagram. Any disagreements surrounding eligibility at either screening stage will be resolved through discussion or consultation with a third reviewer. This two-stage screening process will ensure a thorough and systematic approach to study selection.”*

• P12, L23: how about the age of NDC diagnosis if it was different from the “mean age of sample at study start”.

From our preliminary scoping searches, we do not anticipate identifying many studies in which the mean age at NDC diagnosis and the mean age of sample at study start are different. However, we have now included an additional sentence to for studies where this is the case. It reads *“For studies where the mean age of the exposure and control*

groups are different, where possible we will calculate a pooled mean age of the total sample at the study start and report this in our results. We anticipate that for the majority of studies the mean age of the exposure group at study entry and the mean age at NDC diagnosis will be the same. However, where these differ, we will extract and report both mean ages where possible. Where this is not possible, we will indicate whether the mean age reported in our results refers to the mean age at study entry or the mean age at NDC diagnosis".

• P12, L28: what kind of subgroups do you anticipate?

We included this to account for studies that have looked at NDCs collectively/broadly, but have then also looked at differences according to different forms of NDC (e.g. Autism vs. ADHD). We have now included this as an example to clarify this.

• P13, L21: will the "socio-demographic" info be extracted as well? Suggest stating that in the "Data extraction" section on P12.

Apologies for this oversight, yes where possible we plan to extract information regarding sample ethnicity and sex. We have now included this in the Data Extraction section on P12.

• P13, L29: revise "to influences".

This change has now been made as suggested.

• P14, L8-11: suggest joining this very short paragraph with the previous one.

These two paragraphs have now been combined as suggested.
